# Comparative effectiveness of biological therapies on improvements in quality of life in patients with psoriasis

**DOI:** 10.1111/bjd.15531

**Published:** 2017-10-19

**Authors:** I.Y.K. Iskandar, D.M. Ashcroft, R.B. Warren, M. Lunt, K. McElhone, C.H. Smith, N.J. Reynolds, C.E.M. Griffiths

**Affiliations:** ^1^ Centre for Pharmacoepidemiology and Drug Safety Division of Pharmacy and Optometry School of Health Sciences Faculty of Biology, Medicine and Health The University of Manchester Manchester U.K; ^2^ Dermatology Centre Salford Royal NHS Foundation Trust The University of Manchester Manchester Academic Health Science Centre Manchester U.K; ^3^ Division of Musculoskeletal and Dermatological Sciences School of Biological Sciences Faculty of Biology, Medicine and Health The University of Manchester Manchester U.K; ^4^ NIHR Manchester Biomedical Research Centre Faculty of Biology, Medicine and Health The University of Manchester Manchester U.K; ^5^ Arthritis Research U.K. Centre for Epidemiology Division of Musculoskeletal and Dermatological Sciences Faculty of Biology, Medicine and Health The University of Manchester Manchester U.K; ^6^ St John's Institute of Dermatology Guy's and St Thomas’ NHS Foundation Trust London U.K; ^7^ Institute of Cellular Medicine, Medical School Faculty of Medical Sciences Newcastle University Newcastle upon Tyne U.K; ^8^ NIHR Newcastle Biomedical Research Centre Faculty of Medical Sciences Newcastle University Newcastle upon Tyne U.K; ^9^ Department of Dermatology Royal Victoria Infirmary Newcastle upon Tyne Hospitals NHS Foundation Trust Newcastle upon Tyne U.K

## Abstract

**Background:**

Evidence of the comparative effectiveness of biological therapies for psoriasis on health‐related quality of life (HRQoL) in routine clinical practice is limited.

**Objectives:**

To examine the comparative effectiveness of adalimumab, etanercept and ustekinumab on HRQoL in patients with psoriasis, and to identify potential predictors for improved HRQoL.

**Methods:**

This was a prospective cohort study in which changes in HRQoL were assessed using the Dermatology Life Quality Index (DLQI) and EuroQoL‐5D (EQ‐5D) at 6 and 12 months. Multivariable regression models were developed to identify factors associated with achieving a DLQI of 0/1 and improvements in the EQ‐5D utility score.

**Results:**

In total, 2152 patients with psoriasis were included, with 1239 patients on adalimumab, 517 on etanercept and 396 on ustekinumab; 81% were biologic naïve. For the entire cohort, the median (interquartile range) DLQI and EQ‐5D improved from 18 (13–24) and 0·73 (0·69–0·80) at baseline to 2 (0–7) and 0·85 (0·69–1·00) at 6 months, respectively (*P* < 0·001). Similar improvements were achieved at 12 months. At 12 months, multivariable regression modelling showed that female sex, multiple comorbidities, smoking and a higher DLQI or a lower EQ‐5D utility score at baseline predicted a lower likelihood of achieving a DLQI of 0/1 or improvement in the EQ‐5D. Compared with adalimumab, patients receiving etanercept, but not ustekinumab, were less likely to achieve a DLQI of 0/1. There was no significant difference between the biological therapies in EQ‐5D improvement.

**Conclusions:**

In routine clinical practice biological therapies produce marked improvement in HRQoL, which is influenced by the choice of biological therapy, baseline impairment in HRQoL, lifestyle characteristics and comorbidities. These findings should help inform selection of optimal biological therapy for patients related to improvements in HRQoL.

## Abstract 2

**What's already known about this topic?**

- Evidence of the comparative effectiveness of biological therapies for psoriasis on health‐related quality of life (HRQoL) in routine clinical practice is limited.
- Earlier observational studies were either cross‐sectional, thereby limiting the ability to compare changes in HRQoL, or cohort studies that have not taken into account important clinical factors that could influence treatment response, such as alterations in dosing regimens of biological therapies and the concomitant use of conventional systemic treatments for psoriasis.

**What does this study add?**

- This large prospective cohort study found that in routine clinical practice, the use of biological therapies for psoriasis was associated with marked improvements in HRQoL over 12 months.
- These improvements were influenced by the choice of biological therapy, baseline impairment in HRQoL, lifestyle characteristics and comorbidities.
- Compared with adalimumab, patients receiving etanercept were less likely to achieve a DLQI of 0/1, but there was no significant difference between ustekinumab and adalimumab in the proportion of patients achieving a DLQI of 0/1.
- There was no significant difference between the three biological therapies in level of improvement in the EQ‐5D.

Linked Comment: Finlay. *Br J Dermatol* 2017; **177**:1164–1165.

Psoriasis is a chronic immune‐mediated inflammatory skin disorder, affecting approximately 0·9–8·5% of the population worldwide.^1^ Many patients with psoriasis have moderate‐to‐severe disease that profoundly impacts their emotional wellbeing and health‐related quality of life (HRQoL),^2^, ^3^ with levels of physical and mental disability comparable with those reported for other major medical disorders such as cancer, diabetes and cardiovascular disease.^4^, ^5^ Furthermore, patients with psoriasis have an increased risk of developing comorbid conditions such as psoriatic arthritis (PsA), which can also adversely affect their HRQoL.^6^

Biological therapies have revolutionized the treatment of moderate‐to‐severe psoriasis. The impact of these therapies on HRQoL has been reported in large randomized controlled trials (RCTs).^7^, ^8^, ^9^, ^10^, ^11^, ^12^, ^13^ However, there is a lack of head‐to‐head comparative RCTs assessing the longer‐term impact of these therapies on improvements in HRQoL.^12^, ^14^ Several meta‐analyses have compared the clinical efficacy of different biological therapies for psoriasis, but the results pertain largely to short‐term outcomes and do not always reflect findings in clinical practice.^15^, ^16^, ^17^, ^18^, ^19^, ^20^, ^21^

The effectiveness of biological therapies on disease activity in routine clinical practice has been demonstrated in several prospective observational cohort studies, with up to 80% of patients achieving at least a 75% improvement in the Psoriasis Area and Severity Index (PASI 75).^22^, ^23^, ^24^, ^25^, ^26^, ^27^, ^28^, ^29^ However, evidence of the effectiveness of biological therapies on HRQoL in routine clinical practice is limited to a few observational studies that were either cross‐sectional, thereby limiting the ability to compare changes in HRQoL,^30^, ^31^ or cohort studies that did not take into account important clinical factors that could influence treatment response.^32^, ^33^ Such factors include alterations in dosing regimens of biological therapies over time and the concomitant use of conventional systemic therapies for psoriasis.

The British Association of Dermatologists Biologic Interventions Register (BADBIR) is a U.K. and Republic of Ireland prospective, longitudinal pharmacovigilance register of patients with psoriasis receiving either biological or conventional systemic therapies. Due to its large size, rigorous data collection process, detailed collection of patient demographic characteristics and treatment regimens, and high external validity through participation of 153 dermatology centres,^34^ the register represents an ideal resource to assess the impact of biological therapies on HRQoL in patients with psoriasis in routine clinical practice. In this longitudinal observational study, we examined the comparative effectiveness of adalimumab, etanercept and ustekinumab on improvements in HRQoL in patients with psoriasis, and identified factors associated with these improvements.

## Materials and methods

The BADBIR, established in September 2007, compares a cohort of patients with psoriasis on biological therapies to a similar cohort on conventional systemic therapies. Full details on the design of the BADBIR and the disease characteristics of its participants have been published previously.^34^, ^35^

### Baseline assessment

Baseline data were collected with patient consent and included patients’ demographic characteristics and comorbidities, year of disease onset, standardized measures of health status using self‐reported outcome measures [Dermatology Life Quality Index (DLQI) and EuroQoL‐5D (EQ‐5D)], and detailed information about the patients’ current and previous treatment for psoriasis. Details of the comorbidities were classified using the Medical Dictionary for Regulatory Activities system.^36^

### Follow‐up assessments

Data from patients were collected 6 monthly during the study period. Details of the biological therapies, including any change in the dose or therapy, and start and stop dates, were recorded. Information on any new concomitant systemic therapies for psoriasis and their start and stop dates were also captured. Patient questionnaires also recorded DLQI and EQ‐5D at 6‐ and 12‐month follow‐up.

### Study population

Subjects in this study were selected from the August 2015 data cut‐off. Hence the study time‐frame was from September 2007 to August 2015. Adult patients with chronic plaque psoriasis, receiving adalimumab, etanercept or ustekinumab with follow‐up data of ≥ 6 months were included. The start of observation time was the start date of the index biological therapy (therapy received at enrolment). Only the first biological therapy started during registry participation was analysed. Patients were classified as either biologic naïve or non‐naïve based on their previous exposure to biological therapies prior to registration into the BADBIR. Evaluations were limited to patients who had a valid baseline DLQI (no more than one question left unanswered) and/or EQ‐5D questionnaire (fully completed) recorded within 6 months prior to the start of the index biological therapy and who had another completed questionnaire recorded within 4–8 months and/or 10–14 months (representing the 6‐ and 12‐month follow‐ups, respectively) after the start of the index biological therapy (Fig. S1; see Supporting Information).

### Outcome measures

The DLQI consists of 10 questions evaluating the impact of skin disease on six aspects of HRQoL: symptoms and feelings, daily activities, leisure, work or school performance, personal relationships and treatment.^37^, ^38^ The total score ranges from 0 to 30, with a score of 0–1 indicating no impairment in HRQoL and higher scores indicating greater impairment.^39^ A decrease of ≥ 4 points is considered clinically meaningful.^40^

The EQ‐5D consists of five dimensions that define health: mobility, self‐care, activities, pain/discomfort and anxiety/depression.^41^ Responses to questions yield a utility score that ranges from –0·59–1·00, where 0 represents death, 1 represents full health, and negative values represent health states that are valued as worse than death,^42^ with a change of 0·05 points considered clinically meaningful.^43^

### Statistical analyses

The primary outcome measures were the change in (i) the DLQI total and individual domain scores and (ii) the EQ‐5D profile and utility score from baseline to 6 and 12 months. The proportion of patients who achieved a DLQI of 0/1 at each time point was also assessed. Secondary outcomes included the proportion of patients who achieved an improvement of ≥ 4 and ≥ 0·05 points in the DLQI and EQ‐5D utility scores, respectively, at 6 and 12 months.

Patients were assigned to one of three unique biological cohorts based on their index biological therapy, and recorded as either biologic naïve or non‐naïve. The Wilcoxon signed‐rank test was performed to examine differences in the DLQI total and domain scores and EQ‐5D utility score between baseline and follow‐up results. The McNemar χ^2^‐test was used to examine differences in the proportion of patients reporting any problems in EQ‐5D dimensions between baseline and follow‐up results.

Predictors of change in the EQ‐5D utility score and likelihood of achieving a DLQI of 0/1 were identified at 6 and 12 months using linear and logistic regression models, respectively. An a priori list of covariates was determined to examine potential predictors of response (as presented in Table 5 ). Adalimumab (the most commonly prescribed biological therapy in the BADBIR) was used as the reference biological therapy to which the others were compared.^44^ Concurrent use of methotrexate, ciclosporin and/or other conventional systemic therapies was analysed as a binary variable (ever exposed/never exposed) throughout the study. Dosing patterns of biological therapy were examined using the time‐trend method, which compares the annual cumulative dose patients received to the annual recommended cumulative dose according to product prescribing information.^45^

The DLQI and EQ‐5D analyses were conducted primarily on an intention‐to‐treat basis, using any questionnaire recorded at the appropriate time points after the start of the index biological therapy whether or not the patient was still taking the same biological therapy. Sensitivity analyses in which patients who remained on their index biological therapy when the questionnaires were recorded were also conducted (treatment completers only). Given the large cohort studied and multiple statistical tests, a threshold of *P* ≤ 0·01 was considered to be statistically significant. All calculations were performed using Stata v.14·0 (StataCorp, College Station, TX, U.S.A.).

## Results

In total, 2152 patients with psoriasis (adalimumab 1239, etanercept 517 and ustekinumab 396) were included (Fig. S1; see Supporting Information). The mean (± SD) age of patients, and disease duration were 45·2 ± 12·4 years and 22·4 ± 12·1 years, respectively; 39·4% were female. Mean body mass index (BMI) was 31·1 ± 7·3 kg m^−2^, with 46·9% having a BMI ≥ 30 kg m^−2^. Overall, 73·4% of patients had one or more comorbidities. Baseline demographic and disease characteristics are summarized in Table 1.

**Table 1:** Patient demographic and disease characteristics

	All patients^a^	Etanercept	Adalimumab	Ustekinumab
*n* (%)	2152	517 (24·0)	1239 (57·6)	396 (18·4)
Demographic characteristics				
Age (years), mean ± SD	45·2 ± 12·4	45·1 ± 12·1	44·8 ± 12·4	46·7 ± 12·3
Female	847 (39·4)	217 (42·0)	485 (39·1)	145 (36·6)
BMI category, kg m^−2^				
Nonobese (BMI < 30)	1011 (47·0)	261 (50·5)	582 (47·0)	168 (42·4)
Obese (BMI ≥ 30)	1009 (46·9)	226 (43·7)	590 (47·6)	193 (48·7)
Missing	132 (6·1)	30 (5·8)	67 (5·4)	35 (8·8)
Smoking status				
Never smoked	599 (27·8)	132 (25·5)	358 (28·9)	109 (27·5)
Ex‐smoker	648 (30·1)	131 (25·3)	395 (31·9)	122 (30·8)
Current smoker	532 (24·7)	130 (25·2)	293 (23·7)	109 (27·5)
Missing	373 (17·3)	124 (24·0)	193 (15·6)	56 (14·1)
Psoriatic arthritis/comorbidities				
Psoriatic arthritis	527 (24·5)	129 (25·0)	314 (25·3)	84 (21·2)
No comorbidities	572 (26·6)	140 (27·1)	328 (26·5)	104 (26·3)
1–2 comorbidities	1042 (48·4)	259 (50·1)	623 (50·3)	160 (40·4)
3–4 comorbidities	405 (18·8)	96 (18·6)	222 (17·9)	87 (22·0)
≥ 5 comorbidities	133 (6·2)	22 (4·3)	66 (5·3)	45 (11·4)
Disease				
Disease duration, (years) mean ± SD	22·4 ± 12·1	22·9 ± 12·1	22·3 ± 12·1	22·0 ± 12·1
Age of onset, (years) mean ± SD	22·9 ± 12·9	22·2 ± 12·4	22·6 ± 12·5	24·8 ± 14·5
Baseline DLQI, median (IQR) (*n *= 1804)	18 (13–24)	18 (13–24)	18 (13–23)	19 (13–24)
Baseline EQ‐5D, median (IQR) (*n* = 1618)	0·73 (0·59–0·80)	0·73 (0·52–0·80)	0·73 (0·62–0·80)	0·73 (0·59–0·80)
Unstable psoriasis	268 (12·5)	72 (13·9)	146 (11·8)	50 (12·6)
Medication history				
Biologic naïve	1736 (80·7)	481 (93·0)	1029 (83·1)	226 (57·1)
Concomitant methotrexate^b^	358 (16·6)	82 (15·9)	210 (17·0)	66 (16·7)
Concomitant ciclosporin^b^	149 (6·9)	42 (8·1)	80 (6·5)	27 (6·8)
Concomitant other systemics^b^^,^ c	95 (4·4)	23 (4·5)	45 (3·6)	27 (6·8)

Data are presented as *n* (%) unless otherwise stated. BMI, body mass index; IQR, interquartile range; DLQI, Dermatology Life Quality Index; EQ‐5D, EuroQol‐5D. ^a^Had a complete DLQI (only one question left unanswered) and/or EQ‐5D (no question left unanswered) questionnaire recorded within 6 months prior to the start of the index biological therapy, as well as had another complete DLQI and/or EQ‐5D questionnaire recorded within 4–8 months (representing the 6‐month follow‐up) and/or 10–14 months (representing the 12‐month follow‐up) after the start of the index biological therapy. ^b^Ever used conventional systemic therapies concomitantly with a biological therapy throughout the study period of 12 months. ^c^Includes any of acitretin, fumaric acid esters and hydroxycarbamide.

### Improvements in the Dermatology Life Quality Index

For the entire cohort, the median [interquartile range (IQR)] DLQI improved from 18 (13–24) at baseline to 2 (0–7) at 6 months [median change –13 (–19 to –6); *P* < 0·001] (Table 2). Similar changes were also observed at 12 months. Moreover, the proportion of patients reporting a DLQI of 0/1 increased throughout the study for the whole cohort, from 1·7% at baseline to 45·7% and 48·5% at 6 and 12 months (*P* < 0·001), respectively (Table 3). In addition, 83·6% and 85·9% of the whole cohort achieved an improvement of ≥ 4 points in the total DLQI from baseline at 6 and 12 months, respectively (Table 3). Although 54·3% and 51·5% of the entire cohort did not achieve a DLQI of 0/1 at 6 and 12 months, respectively, 75% of these patients achieved an improvement of ≥ 4 points in their total DLQI from baseline to 6 months; the corresponding figure at 12 months was 81%.

**Table 2:** Values of the Dermatology Life Quality Index (DLQI) total and individual domain scores in patients with psoriasis at different follow‐up times (Intention‐to‐treat analysis)^a^

	All patients	Etanercept	Adalimumab	Ustekinumab
DLQI total score (scale: 0–30)				
Baseline	18 [13–24] (1804)^b^	18 [13–24] (431)	18 [13–23] (1060)	19 [13–24] (313)
6 months	2 [0–7] (1454)c*	4 [1–9] (342)*	1 [0–6] (860)*	2 [0–7] (252)*
Change from baseline to 6 months	−13 [−19 to −6] (1454)	−11 [−17 to −6] (342)	−14 [−20 to −7] (860)	−14 [−19 to −7] (252)
12 months	2 [0–7] (1187)d*	3 [1–9] (293)*	1 [0–6] (689)*	1 [0–6] (205)*
Change from baseline to 12 months	−13 [−19 to −7] (1187)	−11 [−19 to −6] (293)	−14 [−19 to −8] (689)	−14 [−20 to −7] (205)
Symptoms and feelings (scale: 0–6)				
Baseline	5 [4–6] (1757)	5 [4–6] (417)	5 [4–6] (1031)	5 [4–6] (309)
6 months	1 [0–2] (1387)*	2 [1–3] (321)*	1 [0–2] (820)*	1 [0–2] (246)*
12 months	1 [0–2] (1123)*	1 [0–3] (273)*	1 [0–2] (651)*	1 [0–2] (199)*
Daily activities (scale: 0–6)				
Baseline	4 [3–5] (1757)	4 [3–5] (417)	4 [3–5] (1031)	4 [3–5] (309)
6 months	0 [0–2] (1387)*	1 [0–2] (321)*	0 [0–1] (820)*	0 [0–1] (246)*
12 months	0 [0–1] (1123)*	1 [0–2] (273)*	0 [0–1] (651)*	0 [0–1] (199)*
Leisure (scale: 0–6)				
Baseline	4 [2–6] (1757)	4 [2–6] (417)	3 [2–5] (1031)	4 [2–6] (309)
6 months	0 [0–1] (1387)*	0 [0–2] (321)*	0 [0–1] (820)*	0 [0–1] (246)*
12 months	0 [0–1] (1123)*	0 [0–2] (273)*	0 [0–1] (651)*	0 [0–1] (199)*
Work or school (scale: 0–3)				
Baseline	1 [0–2] (1756)	1 [0–2] (416)	1 [0–2] (1031)	1 [0–2] (309)
6 months	0 [0–0] (1386)*	0 [0–1] (321)*	0 [0–0] (820)*	0 [0–0] (245)*
12 months	0 [0–0] (1121)*	0 [0–0] (271)*	0 [0–0] (651)*	0 [0–0] (199)*
Personal relationships (scale: 0–6)				
Baseline	2 [1–4] (1757)	2 [1–4] (417)	2 [1–4] (1031)	3 [1–5] (309)
6 months	0 [0–1] (1387)*	0 [0–2] (321)*	0 [0–0] (820)*	0 [0–1] (246)*
12 months	0 [0–1] (1123)*	0 [0–1] (273)*	0 [0–0] (651)*	0 [0–1] (199)*
Treatment problem (scale: 0–3)				
Baseline	2 [1–3] (1733)	2 [1–3] (414)	2 [1–3] (1013)	2 [1–3] (306)
6 months	0 [0–1] (1355)*	0 [0–1] (317)*	0 [0–1] (797)*	0 [0–1] (241)*
12 months	0 [0–1] (1098)*	0 [0–1] (270)*	0 [0–1] (631)*	0 [0–1] (197)*

^a^Values are presented as median [interquartile range] (number of patients). ^b^47 (2·6%), ^c^67 (4·6%) and ^d^64 (5·4%) patients were included in the analysis of the total DLQI, but were not included in the DLQI individual domain analyses because they only had a total DLQI recorded by the research nurse. *P < 0·001 (calculated for each follow‐up vs. baseline within the same cohort).

**Table 3:** Proportion of patients achieving a Dermatology Life Quality Index (DLQI) of 0/1 and a clinically meaningful improvement of ≥ 4 points from baseline at different follow‐up times (Intention‐to‐treat analysis)

	All patients	Etanercept	Adalimumab	Ustekinumab
Proportion of patients achieving a DLQI of 0/1				
Baseline	31 (1·7) [1804]	7 (1·6) [431]	18 (1·7) [1060]	6 (1·9) [313]
6 months	665 (45·7) [1454]*	101 (29·5) [342]*	445 (51·9) [860]*	118 (46·8) [252]*
12 months	576 (48·5) [1187]*	97 (33·1) [293]*	376 (54·6) [689]*	103 (50·2) [205]*
Proportion of patients achieving a clinically meaningful improvement of ≥ 4 points from baseline				
6 months	1215 (83·6) [1454]	286 (83·6) [342]	716 (83·3) [860]	213 (84·5) [252]
12 months	1020 (85·9) [1187]	248 (84·6) [293]	596 (86·5) [689]	176 (85·9) [205]

Data are presented as *n* (% of patients included in the analysis at that time point) [number of patients included in the analysis]. *P < 0·001 (calculated for each follow‐up vs. baseline within the same cohort).

**Figure 1:**
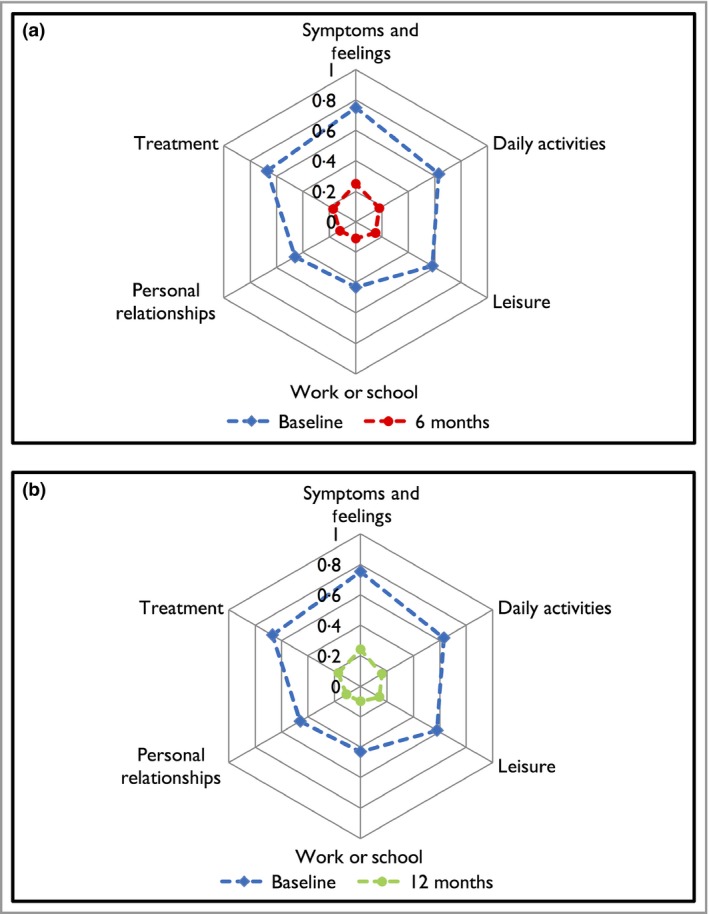
(a) Spider plot of the mean scores at baseline and 6 months in the Dermatology Life Quality Index (DLQI) domains for patients with psoriasis. (b) Spider plot of the mean scores at baseline and 12 months in the DLQI domains for patients with psoriasis. To facilitate direct comparison across the six DLQI domains, the scale was unified to 0–1. To alter the scale for each of the symptoms and feelings, daily activities, leisure and personal relationships domains, the score was divided by 6 (the maximum possible score), and for each of the work or school performance and treatment domains, the score was divided by 3 (the maximum possible score) (Intention‐to‐treat analysis).

Significant improvements were also achieved within 6 months of treatment in all of the six DLQI domains (Fig. 1). Similar response rates were observed at 12 months. The median values of the DLQI total and individual domain scores for each biological cohort, over the 12 month follow‐up period, are presented in Table 2.

### Improvements in the EuroQol‐5D

**Figure 2:**
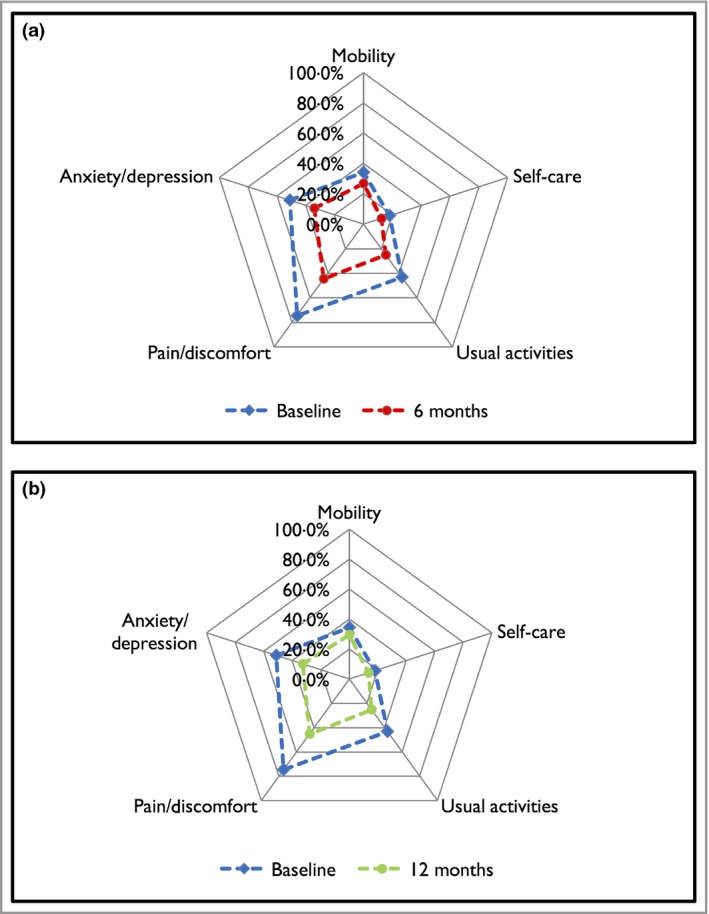
(a) Spider plot of the proportion of patients reporting any problem at baseline and 6 months in the EuroQol‐5D (EQ‐5D) dimensions for patients with psoriasis. (b) Spider plot of the proportion of patients reporting any problem at baseline and 12 months in the EQ‐5D dimensions for patients with psoriasis (Intention‐to‐treat analysis).

The median (IQR) EQ‐5D utility score for the entire cohort improved from 0·73 (0·59–0·80) at baseline to 0·85 (0·69–1·00) at 6 months [median change 0·07 (0–0·273); *P* < 0·001], with 54·2% of patients achieving a clinically meaningful change of ≥ 0·05 points. Similar response rates were found at 12 months (Table 4). The proportion of patients reporting any problems in the EQ‐5D dimensions was significantly reduced from baseline at 6 months. The greatest decrease for the entire cohort was in the pain/discomfort dimension (from 74·5% to 44·6%; *P* < 0·001), whereas the smallest was found in the self‐care dimension (from 18·0% to 12·5%; *P* < 0·001). Similar decreases in dimension scores were also found at 12 months (Fig. 2). The median values of the EQ‐5D utility scores and the proportions of patients reporting any problems in the EQ‐5D dimensions for each biological cohort over the 12‐month follow‐up period are shown in Table 4.

**Table 4:** Values of the EuroQol‐5D (EQ‐5D) utility scores and proportions of patients reporting any problem in the EQ‐5D dimensions in patients with psoriasis at different follow‐up times (Intention‐to‐treat analysis)

	All patients	Etanercept	Adalimumab	Ustekinumab
EQ‐5D utility score, median [IQR] (*n*)				
Baseline	0·73 [0·59–0·80] (1618)	0·73 [0·52–0·80] (391)	0·73 [0·62–0·80] (907)	0·73 [0·59–0·80] (320)
6 months	0·85 [0·69–1·00] (1358)**	0·80 [0·69–1·00] (316)**	0·85 [0·73–1·00] (774)**	0·85 [0·67–1·00] (268)**
Change from baseline to 6 months	0·07 [0·00–0·27] (1358)	0·07 [0·00–0·24] (316)	0·11 [0·00–0·27] (774)	0·07 [0·00–0·24] (268)
12 months	0·85 [0·69–1·00] (1108)**	0·80 [0·69–1·00] (277)**	0·85 [0·71–1·00] (604)**	0·85 [0·66–1·00] (227)**
Change from baseline to 12 months	0·10 [0·00–0·28] (1108)	0·12 [0·00–0·28] (277)	0·11 [0·00–0·27] (604)	0·07 [0·00–0·28] (227)
EQ‐5D dimensions, *n* (%)				
Mobility				
Baseline	555 (34·3)	140 (35·8)	299 (33·0)	116 (36·3)
6 months	365 (26·9)**	88 (27·9)*	190 (24·8)**	85 (31·7)*
12 months	327 (29·5)**	85 (30·7)*	163 (27·0)*	79 (34·8)
Self‐care				
Baseline	291 (18·0)	77 (19·7)	147 (16·2)	67 (20·9)
6 months	170 (12·5)**	41 (13·0)*	81 (10·5)**	48 (17·9)
12 months	149 (13·5)**	32 (11·6)**	74 (12·3)*	43 (18·9)
Usual activities				
Baseline	700 (43·3)	181 (46·3)	381 (42·0)	138 (43·1)
6 months	339 (25·0)**	78 (24·7)**	182 (23·5)**	79 (29·5)**
12 months	279 (25·2)**	70 (25·3)**	141 (23·3)**	68 (30·0)**
Pain/discomfort				
Baseline	1206 (74·5)	296 (75·7)	672 (74·1)	238 (74·4)
6 months	606 (44·6)**	158 (50·0)**	329 (42·5)**	119 (44·4)**
12 months	501 (45·2)**	139 (50·2)**	255 (42·2)**	107 (47·1)**
Anxiety/depression				
Baseline	826 (51·1)	215 (55·0)	453 (49·9)	158 (49·4)
6 months	461 (34·0)**	123 (38·9)**	252 (32·6)**	86 (32·1)**
12 months	361 (32·6)**	101 (36·5)**	187 (31·0)**	73 (32·2)**

IQR, interquartile range. *P < 0·05 (calculated for each follow‐up vs. baseline within the same cohort). **P < 0·001 (calculated for each follow‐up vs. baseline within the same cohort).

### Factors associated with HRQoL improvements

Predictors of being less likely to achieve a DLQI of 0/1 at 12 months included: female sex [odds ratio (OR) 0·71, 95% confidence interval (CI) 0·54–0·93], current smoker vs. never smoked (OR 0·61, CI 0·43–0·87), having any comorbidity vs. having no comorbidities (1–2 comorbidities: OR 0·51, CI 0·37–0·70; 3–4 comorbidities: OR 0·49, CI 0·32–0·75; and ≥ 5 comorbidities: OR 0·39, CI 0·20–0·75); a higher baseline DLQI (for every 1 point increase in the DLQI; OR 0·96, CI 0·95–0·98); concomitant use of methotrexate (OR 0·53, CI 0·38–0·75); receiving etanercept vs. receiving adalimumab (OR 0·39, CI 0·28–0·54); and stopping the index biological therapy (OR 0·35, CI 0·20–0·60) (Table 5).

**Table 5:** Multivariable regression analyses of potential factors associated with achieving a Dermatology Life Quality Index (DLQI) of 0/1 and changes in the EuroQol‐5D (EQ‐5D) utility score at 6 and 12 months (Intention‐to‐treat analysis)

	Achieving a DLQI of 0 or 1^a^		Change in the EQ‐5D utility score^b^	
	6 months	12 months	6 months	12 months
Demographics				
Age (years)^c^	0·97 (0·87–1·09)	1·04 (0·92–1·19)	−0·016 (−0·029 to −0·004)*	–0·015 (–0·027 to –0·002)*
Female	0·91 (0·72–1·15)	0·71 (0·54–0·93)*	−0·019 (−0·046 to 0·008)	0·005 (−0·025 to 0·035)
Obesity status^d^				
Obese (BMI ≥ 30 kg m^−2^)	0·76 (0·60 to 0·96)*	0·78 (0·60 to 1·02)	−0·036 (−0·062 to −0·010)*	−0·012 (−0·041 to 0·018)
Missing	1·00 (0·59 to 1·71)	0·99 (0·58 to 1·69)	0·012 (−0·048 to 0·072)	−0·004 (−0·075 to 0·067)
Smoking status^e^				
Ex‐smoker	0·94 (0·70 to 1·26)	0·77 (0·55 to 1·07)	−0·019 (−0·050 to 0·012)	0·006 (−0·029 to 0·041)
Current smoker	0·83 (0·61 to 1·13)	0·61 (0·43 to 0·87)*	−0·047 (−0·081 to −0·012)*	−0·021 (−0·061 to 0·019)
Missing	0·80 (0·56 to 1·13)	0·66 (0·45 to 0·98)*	−0·014 (−0·053 to 0·025)	0·016 (−0·029 to 0·061)
Comorbidities^f^				
Psoriatic arthritis	1·15 (0·86 to 1·52)	1·09 (0·79 to 1·49)	−0·049 (−0·083 to −0·014)*	−0·077 (−0·120 to −0·034)*
1–2 comorbidities	0·84 (0·64 to 1·10)	0·51 (0·37 to 0·70)*	−0·005 (−0·023 to 0·034)	−0·019 (−0·051 to 0·013)
3–4 comorbidities	0·66 (0·46 to 0·95)*	0·49 (0·32 to 0·75)*	−0·055 (−0·100 to −0·011)*	−0·057 (−0·107 to −0·007)*
≥ 5 comorbidities	0·61 (0·34 to 1·11)	0·39 (0·20 to 0·75)*	−0·158 (−0·232 to −0·084)*	−0·147 (−0·217 to −0·076)*
Disease				
Disease duration (years)^c^	1·14 (1·02 to 1·27)*	1·12 (0·99 to 1·26)	−0·002 (−0·014 to 0·010)	−0·010 (−0·024 to 0·003)
Baseline DLQI	0·98 (0·96 to 0·99)*	0·96 (0·95 to 0·98)*	–	–
Baseline EQ–5D^g^	–	–	0·037 (0·031 to 0·043)*	0·040 (0·034 to 0·046)*
Biologic naïve^h^	1·23 (0·91 to 1·67)	1·17 (0·83 to 1·64)	0·055 (0·016 to 0·094)*	0·014 (−0·025 to 0·053)
Concomitant methotrexate^i^	0·64 (0·46 to 0·88)*	0·53 (0·38 to 0·75)*	−0·036 (−0·073 to 0·002)	−0·009 (−0·046 to 0·029)
Concomitant ciclosporin^i^	0·58 (0·36 to 0·94)*	0·70 (0·42 to 1·15)	0·002 (−0·049 to 0·054)	0·004 (−0·053 to 0·061)
Concomitant other systemicsi,j	0·64 (0·35 to 1·18)	0·60 (0·32 to 1·10)	−0·006 (−0·070 to 0·058)	−0·007 (−0·076 to 0·062)
Dosing pattern^k^				
CD > RCD	1·03 (0·63 to 1·69)	0·74 (0·43 to 1·29)	0·026 (−0·024 to 0·075)	−0·035 (−0·092 to 0·022)
CD < RCD	0·65 (0·38 to 1·12)	0·76 (0·46 to 1·26)	−0·070 (−0·138 to −0·001)*	0·003 (−0·055 to 0·061)
Missing	0·92 (0·57 to 1·48)	0·87 (0·50 to 1·52)	0·048 (−0·004 to 0·099)	0·028 (−0·034 to 0·090)
Biological therapy^l^				
Etanercept	0·37 (0·28 to 0·50)*	0·39 (0·28 to 0·54)*	−0·031 (−0·062 to 0·001)	0·0003 (−0·034 to 0·035)
Ustekinumab	0·86 (0·59 to 1·25)	0·89 (0·57 to 1·37)	−0·026 (−0·069 to 0·016)	−0·030 (−0·078 to 0·019)
Stopped index biological therapy^m^	0·16 (0·08 to 0·32)*	0·35 (0·20 to 0·60)*	−0·077 (−0·151 to −0·002)*	−0·059 (−0·120 to 0·002)

BMI, body mass index; CD, cumulative dose; RCD, annual recommended cumulative dose. ^a^Data are presented as odds ratio (95% confidence interval). ^b^Data are presented as regression coefficient (95% confidence interval). ^c^To evaluate odds ratios and regression coefficients for every 10‐year increase in age and disease duration at enrolment into the register, baseline continuous variables of age and disease duration were transformed to age and disease duration divided by 10. At 6 and 12 months, older age at enrolment (by 10 years) was associated with lower improvement in EQ‐5D values, and longer disease duration (by 10 years) was associated with higher odds of achieving a DLQI of 0/1. ^d^Reference category: nonobese (BMI < 30 kg m^−2^). ^e^Reference category: never smoker. ^f^Reference category: no comorbidities (excluding psoriatic arthritis). ^g^To evaluate regression coefficients for every 0·1 point increase in the EQ‐5D utility score, the baseline continuous variable of EQ‐5D utility score was transformed to EQ‐5D multiplied by 10. At 6 and 12 months, higher baseline EQ‐5D utility score (by 0·1 points) was associated with higher EQ‐5D values. ^h^Reference category: biologic non‐naïve patients. ^i^Included as a yes/no variable, where ‘yes’ = ‘ever used the systemic therapy concomitantly with the biological therapy during the specified time period’ and ‘no’ = ‘never used systemic therapies concomitantly with the biological therapy during the specified time period’. ^j^Includes any of acitretin, fumaric acid esters and hydroxycarbamide. ^k^Reference category CD equal to the RCD; the RCDs according to National Institute for Health and Care Excellence guidelines were:1300 mg (50 mg × 26 weeks) for etanercept; 600 mg [80 mg + (40 mg × 13 weeks)] for adalimumab; and 180 mg (45 mg × 4 doses), or 360 mg (90 mg × 4 doses) if > 100 kg, for ustekinumab at 6 months and 2600 mg (50 mg × 52 weeks) for etanercept; 1120 mg [80 mg + (40 mg × 26 weeks)] for adalimumab; and 270 mg (45 mg × 6 doses), or 540 mg (90 mg × 6 doses) if > 100 kg, for ustekinumab at 12 months. The CD a patient received over the first 6 and 12 months of therapy was calculated as a time‐varying variable taking into consideration any gaps in treatment. ^l^Reference category: adalimumab. ^m^Reference category: continuous users of the index biological therapy. *P < 0·05.

For the change in the EQ‐5D, the multivariable model suggested that with each 10‐year increase in a patient's age there were significantly lower EQ‐5D utility scores at 12 months (regression coefficient –0·015, CI –0·027 to –0·002). Moreover, presence of PsA (regression coefficient –0·077, CI –0·120 to –0·034) and multiple other comorbidities, compared with absence of comorbidities (3–4 comorbidities: regression coefficient –0·057, CI –0·107 to –0·007; or ≥ 5 comorbidities: regression coefficient –0·147, CI –0·217 to –0·076) was significantly associated with lower EQ‐5D utility scores, whereas having a higher baseline EQ‐5D (for every 0·1 point increase in the EQ‐5D utility score; regression coefficient 0·040, CI 0·034–0·046) was significantly associated with higher EQ‐5D response (Table 5).

### Sensitivity analyses

Sensitivity analyses were performed to investigate improvements in the DLQI and EQ‐5D among patients who remained on their index biological therapy at the time the DLQI and/or EQ‐5D questionnaires were recorded. In total, 1294 and 942 patients were included in the DLQI sensitivity analyses at 6 and 12 months, respectively; 1222 and 887 patients were included in the EQ‐5D analyses. Compared with the intention‐to‐treat analyses, a total of 160 and 245 patients were excluded from the DLQI sensitivity analyses at 6 and 12 months, respectively; 136 and 221 patients were excluded from the EQ‐5D analyses because they discontinued their index biological therapy at the time the questionnaires were recorded. Results from the sensitivity analyses did not change the main findings as the magnitude of the improvements observed in the DLQI (Table S1 andS2; see Supporting Information) and EQ‐5D (Table S3; see Supporting Information) were consistent with the main analyses. Likewise, results from the multivariable regression models yielded similar predictors to the main findings (Table S4; see Supporting Information).

## Discussion

This large prospective cohort study found that in routine clinical practice, the use of biological therapies for psoriasis is associated with marked improvements in HRQoL over 12 months. Improvements were influenced by several factors including the choice of biological therapy, baseline impairment in HRQoL, smoking and presence of comorbidities. Compared with adalimumab, patients receiving etanercept, but not ustekinumab, were less likely to achieve a DLQI of 0/1, but there was no significant difference between the three biological therapies in improvement in the EQ‐5D. For the DLQI and the EQ‐5D, a change of 4 and 0·05 points, respectively, correlates with a minimum clinically important difference (MCID).^40^, ^43^ The median differences observed in this cohort study were greater than the MCID at both 6 and 12 months’ follow‐up.

Interestingly, we found that the effectiveness of biological therapies in patients in the BADBIR was less than their reported efficacy in RCTs. For example, the proportion of patients achieving a DLQI of 0/1 in RCTs was 54·4% for etanercept and 57·4% for ustekinumab,^7^, ^46^ compared with 29·5% and 46·8% of patients on etanercept and ustekinumab at 6 months in this cohort study. Furthermore, results from RCTs indicated that EQ‐5D change was between 0·12 and 0·21,^7^, ^47^, ^48^ compared with a change between 0·07 and 0.11 for EQ‐5D at 6 months in the present study. This is likely to be due to differences in demographic and disease characteristics of patients with psoriasis commencing biological therapies in routine clinical practice compared with those enrolled into a clinical trial.^35^

Our findings are in line with those reported by Norlin *et al*.,^32^ who did not find significant differences in change in the EQ‐5D between different biological therapies. However, by comparison, our study has important strengths: the sample size was much larger and we accounted for important clinical factors including smoking and the presence of comorbidities other than PsA. In contrast to our study, Gelfand *et al*.^30^ and Takeshita *et al*.^31^ reported that absolute differences in the DLQI were small and not statistically significant across adalimumab, etanercept and ustekinumab. However, the cross‐sectional design of these studies limits the ability to assess changes in response to therapy. Strober *et al*.^33^ reported that improvements in the DLQI from baseline to 6 and 12 months were significantly better in the ustekinumab group than that in the adalimumab and etanercept groups. However, this study did not adjust for important clinical factors that could influence treatment response, such as dosing adjustments and the concomitant use of conventional systemic therapies with biological therapies.

We have shown that patients on etanercept, but not ustekinumab, were less likely to achieve a DLQI of 0/1 compared with those on adalimumab. This finding aligns with other studies reporting that patients are more likely to discontinue etanercept due to ineffectiveness compared with adalimumab or ustekinumab.^44^ Nevertheless, we found no significant difference between the three biological therapies in improvement in the EQ‐5D. Compared with the EQ‐5D, the DLQI is a dermatology‐specific measure that is more relevant to psoriasis. Hence, the DLQI may have a greater ability to measure specific impairments resulting from the disease and detect smaller changes in health relative to the EQ‐5D.^49^ However, the use of a generic utility instrument (EQ‐5D) allows comparison across different diseases and calculation of quality‐adjusted life years, which will provide valuable data to support cost‐effectiveness analysis.^50^ To our knowledge, this is the first study that has reported on the impact of biological therapies on HRQoL assessed using both a dermatology‐specific measure and a generic utility instrument.

We found that patients who discontinued their biological therapy were less likely to show improvements in HRQoL compared with those who continued therapy. This observation suggests that drug survival is an important proxy marker of effectiveness and real‐world utility.^44^, ^51^

Our study also reports that patients with lower HRQoL (higher DLQI/lower EQ‐5D) at baseline were significantly less likely to achieve a DLQI of 0/1 or show improvement in the EQ‐5D. This finding acknowledges that the ‘cumulative life course impairment’ from living with psoriasis may be a self‐perpetuating social disconnection and failure to achieve ‘full life potential’ in some patients, despite receiving effective therapy.^5^, ^52^ Hence, the devastating impact psoriasis can have on self‐esteem and identity underscores the availability of patient support and psychological treatment as part of routine care.^53^

Consistent with previous studies,^54^ we found that being a current smoker was a predictor of poor improvement in HRQoL, whereas being an ex‐smoker did not predict change in HRQoL, suggesting that smoking could influence response to biological therapies.

As the BADBIR was established primarily as a pharmacovigilance register, there are some limitations to studying the impact of biological therapy on HRQoL that should be considered in interpreting our findings. Firstly, information on patients’ adherence to treatment was not available. Furthermore, as data were collected on a 6‐monthly basis, the study design prevents a more detailed analysis of the time to initial improvement in HRQoL. It has been suggested, in a Swedish study of antitumour necrosis factor use in PsA, that utility improvements occur rapidly (within 2 weeks) and are maintained thereafter.^55^ An inherent limitation in an observational study is nonrandomization that may introduce selection bias, and although this is partially negated by adjustment for clinically relevant covariates, the presence of unmeasured confounders cannot be discounted.

Our results reflect current use of biological therapies for patients with psoriasis in the U.K. and Republic of Ireland, which should be considered in the context of guidelines published by the BAD^56^ and the National Institute for Health and Care Excellence for the management of psoriasis.^57^ Guidelines for the management of psoriasis are also similar in Scotland^58^ and the Republic of Ireland.^59^ Our findings also provide a more solid basis for health economic modelling compared with RCT data due to the greater external validity of the BADBIR.^60^

Further work is required to investigate whether subsequent switching of biological therapies will predict HRQoL changes. Data from patients with PsA in Sweden suggest that improvements in HRQoL during the first and second courses of biological therapies are similar.^55^ Equally important is the need to investigate whether improvements in HRQoL were associated with improvements in disease activity. An earlier study of the PsA cohort within the British Society for Rheumatology Biologics Register found that improvements in HRQoL were significantly associated with improvements in disease activity.^61^

In summary, this large prospective cohort study provides novel insights into the extent of improvement in HRQoL in patients with psoriasis receiving treatment with biological therapies in routine clinical practice, and key determinants of treatment response, which are also of particular importance as they support the concept that lifestyle modifications, including smoking cessation, may enhance the effectiveness of biological therapies. These findings should be considered, along with the other known benefits and risks of biological therapies, when choosing the most appropriate treatment for patients with psoriasis.

## Supporting information


**Fig S1.** Patient selection.
**Table S1.** Values of the Dermatology Life Quality Index total and individual domain scores in patients with psoriasis at different follow‐up times [Treatment completers analysis].
**Table S2.** Proportion of patients achieving a Dermatology Life Quality Index of 0/1 and a clinically meaningful improvement of ≥ 4 points from baseline at different follow‐up times [Treatment completers analysis].
**Table S3.** Values of the EuroQol‐5D (EQ‐5D) utility scores and proportions of patients reporting any problem in the EQ‐5D dimensions in patients with psoriasis at different follow‐up times [Treatment completers analysis].
**Table S4.** Multivariable regression analyses of potential factors associated with achieving a Dermatology Life Quality Index of 0/1 and changes in the EuroQol‐5D utility score at 6 and 12 months [Treatment completers analysis].

**Video S1.** Author video.
