# Supplementary material for: Comparative effectiveness of biological therapies on improvements in quality of life in patients with psoriasis
Source: Br J Dermatol. 2017 Oct 19;177(5):1410–21. doi: 10.1111/bjd.15531 (PMC6487951; doi:10.1111/bjd.15531)
Supplement: Supplementary file 1 — Fig S1. Patient selection. Table S1. Values of the Dermatology Life Quality Index total and individual domain scores in patients with psoriasis at different follow‐up times [Treatment completers analysis]. Table S2. Proportion of patients achieving a Dermatology Life Quality Index of 0/1 and a clinically meaningful improvement of ≥ 4 points from baseline at different follow‐up times [Treatment completers analysis]. Table S3. Values of the EuroQol‐5D (EQ‐5D) utility scores and proportions of patients reporting any problem in the EQ‐5D dimensions in patients with psoriasis at different follow‐up times [Treatment completers analysis]. Table S4. Multivariable regression analyses of potential factors associated with achieving a Dermatology Life Quality Index of 0/1 and changes in the EuroQol‐5D utility score at 6 and 12 months [Treatment completers analysis]. [file BJD-177-1410-s001.docx]

| **Figure S1: Patient selection** | |
| --- | --- |
| *Inclusion criteria* | |
| (1) Recruitment into the biologic cohort (BADBIR) before February, 2015 to allow at least 6 months of follow-up for all patients  Included in the 6 months analysis^††^  (1454)  Included in the 12 months analysis^†††^  (1187)  Included in the DLQI analysis^†^  (1804)  Included in the EQ-5D analysis^‡^  (1618)  Included in the 6 months analysis^‡‡^  (1358)  Included in the 12 months analysis^‡‡‡^  (1108) | 6361 |
| (2) Continuously enrolled and followed up for at least 6 months | 5691 |
| (3) Biologic therapy with either adalimumab, etanercept, or ustekinumab | 5489 |
| (4) Had chronic plaque psoriasis | 5232 |
| (5) Had a DLQI and/or EQ-5D questionnaire at *baseline* recorded within 6 months prior to the start of the index biologic therapy | 3069 |
| (6) Had a DLQI and/or EQ-5D questionnaire recorded within 4-8 months (representing the 6 months of follow-up) and/or 10-14 months (representing the 12 months of follow-up) after the start of the index biologic therapy | 2152^**^ |
|  | |
| Abbreviations: DLQI, Dermatology Life Quality Index; EQ-5D, EuroQol-5D; BADBIR, British Association of Dermatologists' Biologic Interventions Register.  ^**^ This included: 1239 (57.6%) on adalimumab; 517 (24.0%) on etanercept; and 396 (18.4%) on ustekinumab; 1736 (80.7%) were biologic-naïve.  ^†^ The DLQI analysis included patients with a completed DLQI questionnaire (only one question left unanswered) at baseline and at later follow-ups. This included: 1060 (58.8%) on adalimumab; 431 (23.9%) on etanercept; and 313 (17.4%) on ustekinumab. [47(2.6%) patients were included as they had a total DLQI score recorded by the research nurse although all questions of the questionnaire were missing]  ^††^ At 6 months, patients were included if they had a completed DLQI questionnaire at baseline, recorded within 6 months prior to the start of the index biologic therapy; and had another completed DLQI questionnaire recorded within 4-8 months after the start of the index biologic therapy (representing the 6 month follow-up). This included: 860 (59.1%) on adalimumab; 342 (23.5%) on etanercept; and 252 (17.3%) on ustekinumab. [175 patients were excluded due to inaccurately completed DLQI questionnaire; 175 patients were excluded as their DLQI questionnaire was recorded outside the 4-8 months time window] [67 (4.6%) patients were included as they had a total DLQI score recorded by the research nurse although all questions of the questionnaire were missing]  ^†††^ At 12 months, patients were included if they had a completed DLQI questionnaire at baseline, recorded within 6 months prior to the start of the index biologic therapy; and had another completed DLQI questionnaire recorded within 10-14 months after the start of the index biologic therapy (representing the 12 month follow-up). This included: 689 (58.0%) on adalimumab; 293 (24.7%) on etanercept; and 205 (17.3%) on ustekinumab. [215 patients were excluded as they were lost to follow-up; 215 patients were excluded due to inaccurately completed DLQI questionnaire; 187 patients were excluded as their DLQI questionnaire were recorded outside the 10-14 months time window] [64 (5.4%) patients were included as they had a total DLQI score recorded by the research nurse although all questions of the questionnaire were left unanswered]  ^‡^ The EQ-5D analysis included patients with a completed EQ-5D questionnaire (no question left unanswered) at baseline and at latter follow-ups. This included: 907 (56.1%) on adalimumab; 391 (24.2%) on etanercept; and 320 (19.8%) on ustekinumab.  ^‡‡^ At 6 months, patients were included if they had a completed EQ-5D questionnaire at baseline, recorded within 6 months prior to the start of the index biologic therapy; and had another completed EQ-5D questionnaire recorded within 4-8 months after the start of the index biologic therapy (representing the 6 month follow-up). This included: 774 (57.0%) on adalimumab; 316 (23.3%) on etanercept; and 268 (19.7%) on ustekinumab. [129 patients were excluded due to inaccurately completed EQ-5D questionnaire; 131 patients were excluded as their EQ-5D questionnaire was recorded outside the 4-8 months time window]  ^‡‡‡^ At 12 months, patients were included if they had a completed EQ-5D questionnaire at baseline, recorded within 6 months prior to the start of the index-biologic therapy; and had another completed EQ-5D questionnaire recorded within 10-14 months after the start of the index biologic therapy (representing the 12 month follow-up). This included: 604 (54.5%) on adalimumab; 277 (25.0%) on etanercept; and 227 (20.5%) on ustekinumab. [224 patients were excluded as they were lost to follow-up; 126 patients were excluded due to inaccurately completed EQ-5D questionnaire; 160 patients were excluded as their EQ-5D questionnaire were recorded outside the 10-14 months time window] | |

| **Table S1: Values of the DLQI total and individual domain scores in psoriasis patients at different follow-up times^*^[Treatment completers analysis].** | | | | | |
| --- | --- | --- | --- | --- | --- |
|  | All Patients | | Etanercept | Adalimumab | Ustekinumab |
| ***DLQI total score (scale: 0-30)*** | | | | | |
| **Baseline** | | 18 [13-24] (1804)^†^ | 18 [13-24] (431) | 18 [13-23] (1060) | 19 [13-24] (313) |
| **6 months** | | 2 [0-6] (1294)^††^**^**^** | 4 [1-8] (280)**^**^** | 1 [0-5] (774)**^**^** | 2 [0-7] (240)**^**^** |
| **Change from baseline to 6 months** | | -14 [-20;-7] (1294) | -12 [-18;-6](280) | -14 [-20;-8](774) | -14 [-19;-7] (240) |
| **12 months** | | 1 [0-5] (942)^†††^**^**^** | 2 [1-8] (206)**^**^** | 1 [0-4] (554)**^**^** | 1 [0-5] (182)**^**^** |
| **Change from baseline to 12 months** | | -14 [-20;-8] (942) | -12 [-19;-6] (206) | -14 [-20;-9] (554) | -15 [-21;-9](182) |
| ***Symptoms and Feelings (scale: 0-6)*** | | | | | |
| **Baseline** | 5 [4-6](1757) | | 5 [4-6](417) | 5 [4-6](1031) | 5 [4-6](309) |
| **6 months** | 1 [0-2](1233)**^**^** | | 2 [0-2](262)**^**^** | 1 [0-2](737)**^**^** | 1 [0-2](234)**^**^** |
| **12 months** | 1 [0-2](895)**^**^** | | 1 [0-2](195)**^**^** | 1 [0-2](524)**^**^** | 1 [0-2](176)**^**^** |
| ***Daily Activities (scale: 0-6)*** | | | | | |
| **Baseline** | 4 [3-5](1757) | | 4 [3-5](417) | 4 [3-5](1031) | 4 [3-5](309) |
| **6 months** | 0 [0-1](1233)**^**^** | | 1 [0-2](262)**^**^** | 0 [0-1](737)**^**^** | 0 [0-1](234)**^**^** |
| **12 months** | 0 [0-1](895)**^**^** | | 0 [0-2](195)**^**^** | 0 [0-1](524)**^**^** | 0 [0-1](176)**^**^** |
| ***Leisure (scale: 0-6)*** | | | | | |
| **Baseline** | 4 [2-6](1757) | | 4 [2-6](417) | 3 [2-5](1031) | 4 [2-6](309) |
| **6 months** | 0 [0-1](1233)**^**^** | | 0 [0-1](262)**^**^** | 0 [0-1](737)**^**^** | 0 [0-1](234)**^**^** |
| **12 months** | 0 [0-0](895)**^**^** | | 0 [0-2](195)**^**^** | 0 [0-0](524)**^**^** | 0 [0-0](176)**^**^** |
| ***Work and School (scale: 0-3)*** | | | | | |
| **Baseline** | 1 [0-2](1756) | | 1 [0-2](416) | 1 [0-2](1031) | 1 [0-2](309) |
| **6 months** | 0 [0-0](1232)**^**^** | | 0 [0-0](262)**^**^** | 0 [0-0](737)**^**^** | 0 [0-0](233)**^**^** |
| **12 months** | 0 [0-0](893)**^**^** | | 0 [0-0](193)**^**^** | 0 [0-0](524)**^**^** | 0 [0-0](176)**^**^** |
| ***Personal Relationships (scale: 0-6)*** | | | | | |
| **Baseline** | 2 [1-4](1757) | | 2 [1-4](417) | 2 [1-4] (1031) | 3 [1-5] (309) |
| **6 months** | 0 [0-0](1233)**^**^** | | 0 [0-1](262)**^**^** | 0 [0-0](737)**^**^** | 0 [0-1](234)**^**^** |
| **12 months** | 0 [0-0](895)**^**^** | | 0 [0-1](195)**^**^** | 0 [0-0](524)**^**^** | 0 [0-0](176)**^**^** |
| ***Treatment Problem (scale: 0-3)*** | | | | | |
| **Baseline** | 2 [1-3](1733) | | 2 [1-3](414) | 2 [1-3](1013) | 2 [1-3](306) |
| **6 months** | 0 [0-1](1205)**^**^** | | 0 [0-1](260)**^**^** | 0 [0-1](716)**^**^** | 0 [0-1](229)**^**^** |
| **12 months** | 0 [0-1](875)**^**^** | | 0 [0-1](194)**^**^** | 0 [0-1](507)**^**^** | 0 [0-1](174)**^**^** |
| Abbreviations: DLQI, Dermatology Life Quality Index.  *Values are median [interquartile range] (No. of patients)  ^†^47 (2.6%), ^††^61 (4.7%) and ^†††^47 (5.0%) patients were included in the analysis of the total DLQI score, but were not included in the DLQI individual domain analyses because they only had a total DLQI score recorded by the research nurse.  **p<0.0001 and was calculated for each follow-up versus baseline within the same cohort. | | | | | |

| **Table S2: Proportion of patients achieving a DLQI of 0/1 and a clinically meaningful improvement of ≥4 points from baseline at different follow-up times^*^ [Treatment completers analysis]** | | | | |
| --- | --- | --- | --- | --- |
|  | All Patients | Etanercept | Adalimumab | Ustekinumab |
| ***Proportion of patients achieving a DLQI of 0/1*** | | | | |
| Baseline | 31 (1.7%)[1804] | 7 (1.6%)[431] | 18 (1.7%)[1060] | 6 (1.9%)[313] |
| 6 months | 643 (49.7%)[1294]^**^ | 94 (33.6%)[280]^**^ | 433 (55.9%)[774]^**^ | 116 (48.3%)[240]^**^ |
| 12 months | 509 (54.0%)[942]^**^ | 77 (37.4%)[206]^**^ | 334 (60.3%)[554]^**^ | 98 (53.9%)[182]^**^ |
| ***Proportion of patients achieving a clinically meaningful improvement of ≥4 points from baseline*** | | | | |
| 6 months | 1115 (86.2%)[1294] | 239 (85.4%)[280] | 671 (86.7%)[774] | 205 (85.4%)[240] |
| 12 months | 827 (87.8%)[942] | 177 (85.9%)[206] | 491 (88.6%)[554] | 159 (87.4%)[182] |
| * Data presented as n (% of patients included in the analysis at that time-point)[no. of patients included in the analysis]  **p<0.0001 and was calculated for each follow-up versus baseline within the same cohort. | | | | |

| **Table S3: Values of the EQ-5D utility scores and proportions of patients reporting any problem in the EQ-5D dimensions in psoriasis patients at different follow-up times^*^ [Treatment completers analysis]** | | | | |
| --- | --- | --- | --- | --- |
|  | All patients | Etanercept | Adalimumab | Ustekinumab |
| ***EQ-5D utility score*^*^** | | | | |
| **Baseline** | 0.73 [0.59-0.80] (1618) | 0.73 [0.52-0.80] (391) | 0.73 [0.62-0.80] (907) | 0.73 [0.59-0.80] (320) |
| **6 months** | 0.85 [0.73-1.00] (1222)^**^ | 0.85 [0.69-1.00] (266)^**^ | 0.85 [0.73-1.00] (704)^**^ | 0.85 [0.69-1.00] (252)^**^ |
| **Change from baseline to 6 months** | 0.10 [0.00-0.27] (1222) | 0.07 [0.00-0.26] (266) | 0.12 [0.00-0.28] (704) | 0.07 [0.00-0.24] (252) |
| **12 months** | 0.85 [0.69-1.00] (887)^**^ | 0.80 [0.69-1.00] (203)^**^ | 0.85 [0.73-1.00] (482)^**^ | 0.85 [0.69-1.00] (202)^**^ |
| **Change from baseline to 12 months** | 0.11 [0.00-0.28] (887) | 0.11 [0.00-0.31] (203) | 0.12 [0.00-0.28] (482) | 0.07 [0.00-0.24] (202) |
| ***EQ-5D dimensions, n (%)*** | | | | |
| ***Mobility*** | | | | |
| **Baseline** | 555 (34.3%) | 140 (35.8%) | 299 (33.0%) | 116 (36.3%) |
| **6 months** | 309 (25.3%)^**^ | 71 (26.7%)^‡‡^ | 163 (23.2%)^**^ | 75 (29.8%)^‡‡^ |
| **12 months** | 253 (28.5%)^**^ | 59 (29.1%)^‡‡^ | 128 (26.6%)^‡‡^ | 66 (32.7%)^‡‡^ |
| ***Self-Care*** | | | | |
| **Baseline** | 291 (18.0%) | 77 (19.7%) | 147 (16.2%) | 67 (20.9%) |
| **6 months** | 142 (11.6%)^**^ | 33 (12.4%)^‡‡^ | 69 (9.8%)^**^ | 40 (15.9%)^‡‡^ |
| **12 months** | 113 (12.7%)^‡‡^ | 25 (12.3%)^‡‡^ | 54 (11.2%) | 34 (16.8%) |
| ***Usual activities*** | | | | |
| **Baseline** | 700 (43.3%) | 181 (46.3%) | 381 (42.0%) | 138 (43.1%) |
| **6 months** | 279 (22.8%)^**^ | 62 (23.3%)^**^ | 150 (21.3%)^**^ | 67 (26.6%)^**^ |
| **12 months** | 207 (23.3%)^**^ | 52 (25.6%)^**^ | 100 (20.8%)^**^ | 55 (27.2%)^**^ |
| **Pain/Discomfort** | | | | |
| **Baseline** | 1206 (74.5%) | 296 (75.7%) | 672 (74.1%) | 238 (74.4%) |
| **6 months** | 507 (41.5%)^**^ | 123 (46.2%)^**^ | 277 (39.4%)^**^ | 107 (42.5%)^**^ |
| **12 months** | 384 (43.3%)^**^ | 103 (50.7%)^**^ | 191 (39.6%)^**^ | 90 (44.6%)^**^ |
| ***Anxiety/Depression*** | | | | |
| **Baseline** | 826 (51.1%) | 215 (55.0%) | 453 (49.9%) | 158 (49.4%) |
| **6 months** | 391 (32.0%)^**^ | 99 (37.2%)^**^ | 214 (30.4%)^**^ | 78 (31.0%)^**^ |
| **12 months** | 266 (30.0%)^**^ | 68 (33.5%)^**^ | 138 (28.6%)^**^ | 60 (29.7%)^**^ |
| Abbreviations: EQ-5D, EuroQol-5D; n, No. of patients. *Values are median [interquartile range] (No. of patients)  **p<0.0001 and was calculated for each follow-up versus baseline within the same cohort. ‡‡p<0.05 and was calculated for each follow-up versus baseline within the same cohort. | | | | |

| **Table S4: Multivariable regression analyses of potential factors associated with achieving a DLQI of 0/1 and changes in the EQ-5D utility score at 6 and 12 months [Treatment completers analysis]** | | | | |
| --- | --- | --- | --- | --- |
|  | **Achieving a total DLQI score of 0 or 1^†^** | | **Change in the EQ-5D utility score^‡^** | |
|  | 6 months | 12 months | 6 months | 12 months |
| ***Demographics*** | | | | |
| **Age*^1^*** | 0.98(0.87;1.10) | 1.05 (0.91;1.21) | **-0.018 (-0.031;-0.005)^*^** | **-0.015 (-0.030;-0.001)^*^** |
| **Female** | 0.86(0.67;1.09) | **0.65 (0.48;0.87)^*^** | -0.024(-0.053;0.004) | 0.007(-0.027;0.042) |
| ***Obesity status^2^*** | | | | |
| **Obese (BMI ≥30kg/m^2^)** | **0.78 (0.62;0.99)^*^** | 0.75 (0.56;1.01) | **-0.034 (-0.061;-0.007)^*^** | 0.000001(-0.033;0.033) |
| **Missing** | 1.05(0.61;1.81) | 0.96(0.54;1.68) | 0.007(-0.053;0.067) | -0.017(-0.096;0.061) |
| ***Smoking status^3^*** | | | | |
| **Ex-smoker** | 0.97(0.72;1.31) | 0.79(0.55;1.13) | -0.026 (-0.058;0.005) | 0.011(-0.028;0.049) |
| **Current smoker** | 0.87(0.63;1.19) | **0.60 (0.41;0.90)^*^** | **-0.051 (-0.087;-0.016)^*^** | -0.017(-0.061;0.028) |
| **Missing** | 0.84(0.59;1.21) | 0.66 (0.43;1.01) | -0.020(-0.060;0.020) | 0.017(-0.034;0.068) |
| ***Comorbidities^4^*** | | | | |
| **Psoriatic arthritis** | 1.05(0.78;1.40) | 1.28(0.90;1.81) | **-0.043 (-0.079;-0.007)^*^** | **-0.063 (-0.107;-0.018)^*^** |
| **1-2 comorbidities** | 0.82(0.62;1.10) | **0.46 (0.32;0.65)^*^** | -0.0001(-0.029;0.028) | -0.025(-0.059;0.008) |
| **3-4 comorbidities** | **0.66 (0.45;0.96)^*^** | **0.41 (0.25;0.65)^*^** | **-0.062 (-0.109;-0.016)^*^** | **-0.066 (-0.121;-0.012)^*^** |
| **≥5 comorbidities** | 0.65(0.36;1.20) | **0.33 (0.15;0.68)^*^** | **-0.165 (-0.244;-0.086)^*^** | **-0.178 (-0.257;-0.098)^*^** |
| ***Disease*** | | | | |
| **Disease duration*^1^*** | **1.14 (1.02;1.27)^*^** | **1.16 (1.02;1.33)^*^** | -0.001 (-0.012;0.013) | -0.013(-0.029;0.002) |
| **Baseline DLQI** | **0.98 (0.97;0.99)^*^** | **0.96 (0.95;0.98)^*^** | - | - |
| **Baseline EQ-5D*^5^*** | - | - | **0.037 (0.031;0.043)^*^** | **0.039 (0.032;0.045)^*^** |
| **Biologic Naive^6^** | 1.15(0.84;1.57) | 1.36(0.94;1.98) | **0.062 (0.021;0.102)^*^** | 0.036 (-0.008;0.080) |
| **Concomitant methotrexate^7^** | **0.64 (0.46;0.90)^*^** | **0.47 (0.33;0.68)^*^** | -0.033(-0.071;0.004) | -0.011(-0.053;0.030) |
| **Concomitant ciclosporine^7^** | 0.62(0.37;1.01) | 0.79(0.45;1.41) | 0.017(-0.037;0.070) | 0.012(-0.047;0.071) |
| **Concomitant other systmics^7,8^** | 0.66(0.36;1.23) | 0.62(0.31;1.22) | 0.015(-0.052;0.082) | -0.017(-0.089;0.056) |
| ***Dosing pattern^9^*** | | | | |
| **CD > RCD** | 1.10(0.66;1.82) | 0.83(0.46;1.50) | 0.032(-0.019;0.083) | -0.036(-0.096;0.023) |
| **CD < RCD** | 0.66(0.37;1.18) | 0.61(0.34;1.10) | -0.066(-0.144;0.012) | -0.016(-0.083;0.050) |
| **Missing** | 0.91(0.56;1.48) | 0.93(0.52;1.68) | 0.039(-0.013;0.092) | 0.040(-0.023;0.103) |
| ***Biologic therapy^10^*** | | | | |
| **Etanercept** | **0.36 (0.27;0.49)^*^** | **0.35 (0.25;0.51)^*^** | **-0.041 (-0.073;-0.008)^*^** | -0.009(-0.050;0.032) |
| **Ustekinumab** | 0.83(0.57;1.21) | 0.96 (0.60;1.53) | -0.023(-0.066;0.021) | -0.020 (-0.070;0.029) |
| Abbreviations: BMI, body mass index; PASI, psoriasis area and severity index; DLQI, dermatology life quality index; EQ-5D, EuroQol-5D; CD, cumulative dose; RCD, annual recommended cumulative dose.  † Data presented as odds ratio (95% confidence interval). ‡ Data presented as regression-coefficients (95% confidence intervals).*p-value<0.05, shown in bold.  ^1^ To evaluate odds ratio and regression-coefficients for every 10 years increase in age and disease duration at enrolment into the register, baseline continuous variable of age and disease duration were transformed to age and disease duration divided by 10. At 6 and 12 months, older age at enrolment (by 10 years) were associated with lower improvement in the EQ-5D values and longer disease duration (by 10 years) were associated with higher odds of achieving a DLQI of 0/1.  ^2^ Reference category: non-obese (BMI <30kg/m^2^); ^3^ Reference category: never smoker; ^4^Reference category: no comorbidities (excluding psoriatic arthritis).  ^5^ To evaluate regression-coefficients for every 0.1 point increase in the EQ-5D utility score, baseline continuous variable of EQ-5D utility score was transformed to EQ-5D multiplied by 10. At 6 and 12 months, higher baseline EQ-5D utility score (by 0.1 points) was associated with higher EQ-5D values.  ^6^ Reference category: biologic non-naive patients.  ^7^ Included as a yes/no variable, where yes=ever used the systemic therapy concomitantly with the biologic therapy during the specified time period and no= never used systemic therapies concomitantly with the biologic therapy during the specified time period.  ^8^Includes any of acitretin, fumaric acid esters and hydroxcarbamide.  ^9^ Reference category CD equal to the RCD; The RCDs according to NICE guidelines were:1300mg (50mg x 26 weeks) for etanercept; 600mg (80mg + (40mg x 13 weeks)) for adalimumab and; 180mg (45mg x 4 doses),or 360mg (90mg x 4 doses) if >100Kg, for ustekinumab at 6 monthsand2600mg (50mg x 52 weeks) for etanercept; 1120mg (80mg + (40mg x 26 weeks)) for adalimumab and; 270mg (45mg x 6 doses),or 540mg (90mg x 6 doses) if >100Kg, for ustekinumab at 12 months. The CD that a patient received over the first 6 and 12 months of therapy was calculated as a time-varying variable taking into consideration any gaps in treatment.  ^10^ Reference category: adalimumab. | | | | |
